# AGING IN LATIN AMERICA: HEALTH, DISPARITIES, AND OUTCOMES AMONG OLDER ADULTS

**DOI:** 10.1093/geroni/igae098.0310

**Published:** 2024-12-31

**Authors:** Margarita Maria Osuna, Rafael Samper-Ternent

**Affiliations:** Chair; Discussant

## Abstract

Older adults in Latin America are living in contexts of high levels of poverty, low education, poor health literacy and reduced healthcare access. This symposium is focused on heath-disparities among older adults living in Brazil, Costa Rica, Mexico, Puerto Rico, and Colombia. The symposium explores connections between aging and health such as longevity, cognition, disability, and wellbeing in the context of Latin America, aiming to shed light on health-related dimensions and disparities among older Latinos. Using data from the Brazilian Longitudinal Study of Aging, Gomes examines the relationship between hearing loss and cognitive decline. Using the longitudinal Puerto Rican Elderly Health Conditions Project and the Puerto Rico Contextual Data Resource, García, examines how the relationships between racial identity, neighborhood-level racial density, and neighborhood-level socioeconomic status are related to the probability of experiencing all-cause mortality among island-dwelling Puerto Ricans. Using four nationally representative surveys of older adults in Latin America, Osuna studies how disability prevalence varies among older adults in Mexico, Costa Rica, Colombia and Brazil. Lastly, Saenz studies evaluates how level of exposure to a major drought Mexico is related with health among older adults, for this study they use data from the Mexican Health and Aging Study. Results underline what populations in Latin American have increased risk of experiencing poorer health outcomes and disparities that may exist. The findings highlight the importance of understanding the conditions under which Latin American older adults are aging and the implications this can have in the future.

